# Assessment of Veterinary Drug Availability, Storage Conditions, and Handling Practices in and Around Nekemte Town, Southwestern Oromia, Ethiopia

**DOI:** 10.1155/vmi/7813053

**Published:** 2025-09-03

**Authors:** Abibo Wondie Mekonen, Natinael Senbato, Melaku Getahun Feleke, Merawi Kindu Birhanie, Yesuneh Tefera Mekasha

**Affiliations:** ^1^Department of Veterinary Pharmacy, Pharmaceutical Supply Chain Management, College of Veterinary Medicine and Animal Sciences, University of Gondar, Gondar, Ethiopia; ^2^Department of Veterinary Medicine, College of Veterinary Medicine and Animal Sciences, University of Gondar, Gondar, Ethiopia; ^3^Department of Veterinary Pharmacy, Pharmaceutical Analysis and Quality Assurance, College of Veterinary Medicine and Animal Sciences, University of Gondar, Gondar, Ethiopia; ^4^Department of Veterinary Clinical Studies, Veterinary Obstetrics and Gynecology, College of Veterinary Medicine and Animal Science, University of Gondar, Gondar, Ethiopia; ^5^Department of Veterinary Pharmacy, Pharmaceutical Quality Assurance and Regulatory Affairs, College of Veterinary Medicine and Animal Sciences, University of Gondar, Gondar, Ethiopia

**Keywords:** availability, Ethiopia, KAP, Oromia region, storage conditions, veterinary drugs

## Abstract

Animal diseases pose a significant threat to both animal welfare and productivity, making veterinary drugs essential for their prevention, treatment, and control. In developing countries including Ethiopia, several challenges such as limited drug availability, inadequate storage facilities, improper handling, and insufficient knowledge among veterinary professionals impede the delivery of effective veterinary health services. This study aimed to assess the availability of veterinary drugs, storage conditions, and the knowledge, attitudes, and practices (KAP) of veterinary professionals regarding the safe management of these drugs. A cross-sectional study was conducted across 46 veterinary facilities (34 government clinics and 12 private pharmacies), involving 170 veterinary professionals who participated through structured questionnaires and observational checklists. Data were analyzed using SPSS and Microsoft Excel. The study revealed suboptimal drug availability, with multivitamins being the most commonly available (84.8%), followed by anthelmintic (72.8%) and antibacterial (69.6%), while antifungals were the least available (18.3%). Storage conditions were inadequate in both government clinics (64.5%) and private retailers (65.6%), falling below the acceptable threshold of over 80% for good storage practice. In terms of KAP, 52.4% of professionals demonstrated low knowledge, 77.6% held negative attitudes, and 50.6% exhibited poor practices related to the safe handling and storage of veterinary drugs. These results indicate critical gaps in drug availability, storage standards, and professional competence. Addressing these challenges requires coordinated action from the Regional Livestock and Agricultural Bureau and the Ethiopian Agricultural Authority (EAA), with emphasis on strengthening supply chains, upgrading storage infrastructure, and implementing targeted training programs for veterinary professionals.

## 1. Introduction

Animal diseases have a significant effect on animal welfare and productivity. Nonetheless, compared to a vast and widely accessible animal supply, Ethiopia produces less livestock productivity [1, 2]. Both infectious and noninfectious diseases pose a threat to their performance and future, endangering the country's livestock productivity [3, 4]. Veterinary pharmaceuticals (VPs) play a significant role in animal disease prevention, treatment, and control. VPs, sometimes known as veterinary medications, are compounds used for prevention, treatment, and control of diseases [5, 6].

In the past, industrialized nations like the USA and several European nations were the main users of antimicrobials in food animals. Antimicrobial usage in livestock has increased dramatically in recent years in developing countries in South and Southeast Asia, including Bangladesh, Bhutan, India, Indonesia, Myanmar, Nepal, Sri Lanka, and Thailand. In Bangladesh, approximately 94.16% of poultry farmers use antibiotics in their operations [7–10]. These days, antibiotics used in food animals account for 80% of the country's yearly antibiotic use in the United States, and about 80% of all food-producing animals and birds take treatment for all or most of their lives [9, 11]. In developing countries, the demand for livestock products has increased significantly with the advancement of veterinarian medical utilizations [12]. In Ethiopia, currently large amounts of antimicrobials, anthelmintic, and antiprotozoal are used for the treatment, prophylaxis, and control of animal disease in the clinical practice of veterinary medicine throughout different borders of the country [12–14].

The lack of access to veterinary drug is the problem of sub-Saharan African (SSA) countries and remains an issue for many farmers [15]. The provision of effective animal health care and the maintenance of quality veterinary medications are dependent on the presence of uninterrupted drug supply, the practices of good pharmaceutical handling, and storage conditions [15–18]. The quality, safe, and effective veterinary drugs have become an extremely important issue both in international public health concerns in addition to animal welfare and productivity. In developing countries, lack of access and the reduction of quality and shelf life of pharmaceutical products typically occur due to indiscriminate, improper handling practices, and poor management of VPs following the storage conditions and KAP of veterinary professionals in relation to the safe handling of drugs [15, 16, 19, 20].

Inappropriate use of veterinary medications will not only lower the standard of animal health care but also increase the likelihood that people may develop antimicrobial resistance (AMR) [19, 20]. Numerous researchers have found that the livestock subsector, particularly the animal healthcare services, is vulnerable to a range of issues related to the VP supply chain, including lack of access to veterinary drugs, improper drug handling during purchase, irrational drug use, illegal marketing, low-quality medicines, a lack of waste management practices, low rates of adherence to rules and policies, a lack of qualified and trained staff members, and poor practices regarding the rational use and the safe handling of veterinary drugs are some of the issues [5, 13, 20–24]. According to a study done in Nigeria, Africa, cattle diseases that may have been prevented have cost the continent $4 billion losses [20]. The study conducted in Dares Salaam, Tanzania, revealed that poor record keeping and the lack of guidelines on the appropriate disposal of medicines are the factors that affect drug handling and management [25]. Another study conducted on the veterinary drug supply chain in Uganda revealed that nearly ninety percent (90%) of drug retailers and veterinary practitioners did not receive specialized training in veterinary medicine handling and storage management [26]. A study carried out in Ethiopia on the quality of veterinary drugs during postmarketing surveillance, reregistration, consignment checking, and preregistration indicates that about 12 (1.3%) of the examined products had defects in their appearance, packaging, or labeling, and 8.2% of the examined veterinary medication samples had poor quality labels [27]. Another studies also showed that the production, importation, distribution, and use of biological products and veterinary medications are not adequately controlled or regulated with regard to efficacy, safety, and quality [6, 19]. A study conducted in the Amhara region, Ethiopia, showed that the performance of veterinary health facility's veterinary drug storage conditions and management practices in the study facilities was only about 48.3%, which was considerably poor [17].

Furthermore, a study in Afar indicated that about 63.9% of the respondents did not have enough knowledge on the safe handling and management of veterinary drugs [16]. However, yet knowledge, little is known in the Oromia region yet about the accessibility of veterinary drugs, storage management practices, and the KAP of professionals on the safe handling of veterinary drugs in the study area. Hence, the present study is conducted to assess the veterinary drug availability, storage conditions, and the KAP of veterinary professionals working at governmental veterinary clinics and private drug venders in and around Nekemte town.

### 1.1. Research Questions and Research Hypothesis

The study aimed to address its objectives through three primary research questions: (1) Is there a significant difference in the availability of veterinary drugs between governmental clinics and private veterinary pharmacy retailers? (2) Do veterinary professionals' levels of knowledge significantly influence their drug handling and storage practices? (3) Are the storage conditions of veterinary drugs in the surveyed facilities significantly below the acceptable threshold for good storage practices (> 80%)? To explore these questions, the study proposed the following null hypotheses: (1) There is no significant difference in the availability of veterinary drugs between governmental clinics and private veterinary pharmacy retailers; (2) veterinary professionals' levels of knowledge do not significantly influence their drug handling and storage practices; and (3) the storage conditions of veterinary drugs in the surveyed facilities are not significantly below the acceptable good storage practice threshold of 80%.

## 2. Materials and Methods

### 2.1. Study Area

This study was conducted in and around Nekemte, southwestern Oromia, Ethiopia, from January to August 2024. Nekemte is located in the western part of Oromia, Ethiopia, 331 km from Addis Ababa, the capital city. It lies at a latitude of 9°04′ North and a longitude of 36°30′ East, at an altitude ranging from 750 to 3178 m above sea level.

Administratively, there are 17 districts, with many kebele in each districts, and 7 city administrations in and around Nekemte. For this study, all 17 districts were found in and around Nekemte, namely, the (Diga, Gidda Ayyana, Gobbu Sayyo, Gudaya Bila, Guto Gida, Haro Limu, Leqa Dulacha, Ebantu, Sasiga Sibu Sire, Wayu Xuqa, Guto Wayu, Nunu Qumba, Wama Haqalo, Bonaya Boshe, Kiramu, Lummu, Jimma Arjo, and City of Nekemte). According to the 2023 report acquired from the Oromia regional state livestock resource development office, in the study area, there are a total of 170 veterinary facilities (17 governmental woreda veterinary clinics, 121 governmental kebele veterinary clinics, 20 private veterinary clinics, and 12 private veterinary retailers) serving to care for over 18 million livestock populations. The governmental veterinary clinics, private veterinary clinics, and drug retailers were found in the woreda main city and its kebele level.

### 2.2. Study Design

A cross-sectional study design was carried out, and a facility survey was conducted at the selected veterinary health facilities using self-administered questionnaires and observational checklists with the aim of assessing the availability of commonly used veterinary drugs, evaluating the storage area of veterinary drugs, and KAP of veterinary professionals toward the proper and safe handling of veterinary drugs in veterinary health facilities in and around Nekemte, Oromia regional state, Ethiopia.

### 2.3. Population of the Study

#### 2.3.1. Source Population

The source population for this study was all the governmental veterinary clinics, all private veterinary clinics, all private drug retailers, and all employees who are actively working in the clinics, and drug shops in and around Nekemte.

#### 2.3.2. Study Population

To assess the veterinary drug availability and storage conditions, all governmental district veterinary clinics and all private veterinary drug retailers were included in the study population. The selection of these facilities was due to the fact that district clinics and private pharmacy retailers have a chance to hold various types of VP products, whereas governmental veterinary clinics found at the kebele level were selected randomly from each district. To assess the KAP, professionals who are working in the selected veterinary health facilities participated.

### 2.4. Eligibility Criteria

#### 2.4.1. Inclusion and Exclusion Criteria

District and kebele governmental veterinary clinics and private veterinary drug retailers were included to assess drug availability and evaluation of drug storage conditions. The reason for the inclusion of these facilities was that, in practical terms, these facilities have a chance to hold various types of VP products, whereas private veterinary clinics were excluded, as they do not have a grant to hold pharmaceutical stock. To assess the KAP toward the safe handling of veterinary drugs, those professionals who were participating in veterinary drug prescription, purchasing, distribution, and dispensing activities were included in the study.

### 2.5. Sample Size and Sampling Techniques

#### 2.5.1. Sample Size Determination

For this study, the formula for calculating sample sizes when the proportion is the parameter of research, which was developed by Cochran (1963), was applied, and a 90% confidence level with a 10% margin of error was used. Using this formula, a total of 46 veterinary health facilities were selected as a study sample from a refined population of 150 facilities (districts and kebele governmental veterinary clinics and 12 private veterinary drug shops) in the study area that fulfill the inclusion criteria. Specifically, the study sample size therefore consisted of 17 district veterinary clinics, 17 kebele veterinary clinics from the governmental sector, and 12 veterinary drug retailers from the private sector.

The general formula for calculating the sample size is

(1) $$\begin{array}{c} n_{0}=\frac{Z2*p*q}{e2},\\ n_{0}=\frac{{\left(1.64\right)}^{2}\times 0.5\left(1-0.5\right)}{{\left(0.1\right)}^{2}}, \end{array}$$

*n*_0_ = 67. Where *n*_0_ is the sample size required, *Z* is the *Z* value (1.64 for 90% confidence level), and *p* is the estimated prevalence of the indicator; the product of [*p*] and [*q*] is maximized when *p* = 0.5.

Therefore, when the prevalence is unknown, 0.5 should be used, and *e*2 = the 10% margin of error used in estimating the prevalence, *e*2 = 0.1. However, the sample size (*n*_0_) was adjusted as *n*: This adjustment can substantially reduce the necessary sample size for small populations and is also called the population correction factor [28].

(2) $$\begin{array}{c} n=\frac{n_{0}}{\left[1+\left\{\left(n_{0}-1\right)/N\right\}\right]}, \end{array}$$

where *n* = the adjusted new sample size; *N* = the population size (150); *n*_0_ = the sample size obtained from the general formula.

(3) $$\begin{array}{c} n=\frac{67}{\left[1+\left\{\left(67-1\right)/150\right\}\right]},\\ n=46\;\left(\text{veterinary health facilities}\right). \end{array}$$

### 2.6. Sampling Techniques

To select study units, first all clinics and private drug retailers in and around Nekemte were identified, and then veterinary health facilities that did not fulfill the inclusion criteria were excluded. After that, veterinary health facilities were stratified as governmental and private sectors. Finally, governmental veterinary clinics and private veterinary drug retailers were selected using purposive sampling techniques based on the inclusion criteria set for this study (they hold various types and huge stocks of pharmaceutical products). To address objectives 1 and 2, a total of thirty-four (*n* = 34) governmental district veterinary clinics, which are selected purposefully, and seventeen (*n* = 17) governmental kebele clinics, which were randomly selected from each district, were selected from the governmental sector, and twelve (*n* = 12) private drug retailers' shops were proposed purposively from the private sector. Accordingly, a total of 46 veterinary health facilities were included. To address objective 3, participants who have a direct involvement in veterinary drug purchasing, prescriptions, handling, storage, and distribution in the selected facilities were included. Accordingly, a total of 170 participants (veterinary clinicians, veterinary drug dispensers, veterinary pharmacy shop owners, and veterinary drug and input supply distributor officers) participated.

### 2.7. Data Collection Tools and Procedures

#### 2.7.1. Data Collection Tools

To collect the necessary data, the logistic indicator assessment tool (LIAT) developed by USAID/Deliver project [29], the directives of the Veterinary Drug and Fee Administration Control Authority (VDFACA) and a list of veterinary drugs for Ethiopia published in 2002 were adapted and customized to the local context. Moreover, in order to improve the study and strengthen the findings, data collection tools from various research studies and related articles were also referred to in the adoption [5, 14, 16, 17, 19]. Accordingly, self-administered questionnaires and observational checklists were used to collect the data.

### 2.8. Data Collection Procedures

A consent form was prepared, and communication was done with respondents to get their consent. Once their consent was known, the prepared questionnaires were distributed to each participant, and then the quantitative data were collected using a self-administered questionnaire and observational checklists. Structured questionnaires were used to collect KAP data, with participants completing them on their own. To collect data on the availability and storage conditions, a survey was done at veterinary health facilities using observational checklists. The district veterinary clinic's veterinary health input supply and distribution officer, veterinary drug store and control personnel, drug dispensers, and veterinary drug assistant storekeepers from the sampled facilities were participated in filling out the self-administered questionnaire. The data that were collected using an observational checklist were completed by the researcher through direct visits to the sampled facilities.

### 2.9. Data Quality Assurance

The study questionnaires were shaped after reviewing various literature sources as well as a veterinary drug management and regulatory guideline. The quality of the data was ensured by implementing multiple strategies. First, the information was coded and reviewed for correctness, consistency, and missing information. To ensure the data's quality, several strategies were implemented. Initially, the data were coded and checked for accuracy, consistency, and omissions. It was then prepared for analysis using validated data collection forms from the selected study areas. A data collector received 2 days of training on the tools and procedures used in data collection before beginning the actual data collection. A pilot study was conducted to pretest the research instrument, which involved distributing the questionnaires to 20 veterinary drug professionals working in the selected study area. The findings from the pilot study were used to make necessary edits to the questionnaire, improving its clarity and flow. Finally, the investigators double-checked the quality of the data to ensure its accuracy.

### 2.10. Data Processing and Analysis Methods

After the data were collected, coding, entry, error editing, and analysis were done through the utilization of Statistical Package for Social Sciences (SPSS) Version 26 and Microsoft Excel 2010. Descriptive statistics were generated, along with the usage of graphs, tables, and numerical summaries to present the results. Findings from the observational checklist, which is the percentage of drug availability at each facility of governmental veterinary clinics and private veterinary drug retailers, were calculated as the average of the number of yes responses for each drug in the checklist to the number of veterinary facilities assessed, whereas the storage conditions at governmental veterinary clinics and private veterinary drug retailers were calculated as the average of the number of yes responses in the checklist to the number of standard storage conditions in the checklist.

A. Percentage of veterinary drug availability was calculated as
  (4) $$\begin{array}{c} \frac{\text{The number of yes responce for each item}}{\text{The number of veterinary facilities surveyed }}\times 100. \end{array}$$
B. The percentage of storage condition was calculated as

(5) $$\begin{array}{c} \frac{\text{The number of yeas responce in the checklist}}{\text{Number of standared criteria used in the checklist}}\times 100. \end{array}$$

The interpretation was made based on the recommendation of a previous researcher [17, 30]. Accordingly, facilities that fulfill at least 80% of the criteria presented in the checklist can be considered acceptable and said to have good storage conditions. For analysis of the KAP data, the data were coded the correct answer as 1, and the wrong answer as 0, for each item. The respondent's level of knowledge, attitude, and practices (KAP) was assessed using a total of 25 questions; each level (KAP) is categorized as “high/low,” “negative/positive,” and “good/poor,” respectively, using the individual score and the principle used by different researchers for the KAP study [31–33].

Participants' knowledge about veterinary drug handling practices and storage conditions was assessed using 11 questions. Each knowledge question had “true,” “false” possible responses. The level of the respondent's knowledge was categorized into two levels based on Bloom's cutoff point and by different researchers for the KAP study [34, 35]. Accordingly, if the score was ≥ 50% (≥ 7 points out of 11 questions) as high level, and below this, it was considered as a low level of knowledge. Attitude toward the safe handling practices and storage of veterinary products was assessed using seven questions. Responses to questions related to attitude were graded on a 2-point Likert scale ranging from “1” for agree to 2 and for disagree. The level of attitude was categorized as positive and negative using Bloom's cutoff point; accordingly, if the score was ≥ 50% (≥ 5.1 points out of 7 questions) as a positive attitude and if the score was < 50% (< 5.1 points out of 7 questions) as a negative attitude. Regarding practices, the practices of veterinary professionals' toward safe handling of veterinary drugs were assessed using seven questions. The previous rating scale was used to assess the responses. The scores for measuring the practice of veterinary safe handling of veterinary drugs are classified into two levels: good practices and poor practices. The levels of practice were good if they were ≥ 50% (6.06 points out of 7 questions).

## 3. Results

### 3.1. Sociodemographic Characteristics of the Study Participants

A total of 46 veterinary health facilities (*n* = 34 (73.9%) governmental veterinary clinics and *n* = 12 (26.1%) private veterinary pharmacy retailers) were surveyed for this study. A total of 170 study participants participated in filling out the self-administered questionnaire. Among them, the majority, 106 (62.4%), were male. Regarding their position, the majority 100 (58.8%) were veterinary clinicians, followed by drug dispensers 40 (23.5%). From the respondents, the majority 88 (51.8%) were BVS in their level of qualification, followed by advanced animal health 50 (29.4%) (Table 1).

### 3.2. Availability of Common Veterinary Drugs Across Surveyed Health Facilities

The survey data show that antibacterial and anthelminthic drugs are generally well stocked in most veterinary health facilities, with oxytetracycline (both 10% and 20% formulations) and albendazole (2500 and 300 mg) being the most available drugs, each present in over 75% of the facilities. Other important antibacterial like Penstrep and sulfonamides also show moderate availability, ranging from 63% to nearly 70%. However, gentamycin stands out as having significantly lower availability, present in only about a quarter of the surveyed facilities. Similarly, among anthelminthics, drugs such as Ivermectin injection and Fenbendazole sachets are widely available, but others such as Tetramisole show a reduced presence, with availability closer to 60% (Table 2).

In contrast, availability is notably lower for antiprotozoal, antifungal, mineral, and indigestion-related products. For instance, essential antiprotozoals like isometamidium and Amprolium hydrochloride have limited presence, with availability rates of only 21.7% and 39.1%, respectively. Antifungal ketoconazole and copper sulfate minerals are scarcely available, found in less than 20% of facilities. Additionally, antiseptics like alcohol and iodine solutions are relatively common, though potassium permanganate is less so. Vitamins, particularly multivitamins, are widely stocked, being available in nearly 85% of facilities. This distribution suggests a stronger focus on antibacterial and anthelminthic drugs in veterinary services, while protozoal treatments and certain supportive therapies might be underrepresented, potentially impacting comprehensive animal healthcare delivery.

**Figure 1:**
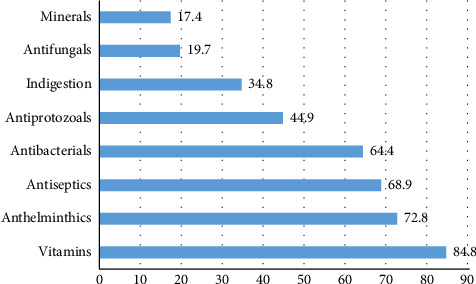
Percentage availability of veterinary pharmaceuticals by class categories.

The availability of VPs varies significantly across different drug categories. Vitamins are the most accessible, with an average availability of 84.8%, reflecting their widespread use in animal health and nutrition. Anthelminthic (72.8%), antiseptics (68.9%), and antibacterial (64.4%) also show relatively high availability, likely due to their essential role in parasite control, wound management, and bacterial infection treatment (Figure 1). In contrast, antiprotozoals (44.9%) and indigestion medications (34.8%) are less readily available, possibly because they target conditions that are more specific. The lowest availability was seen in antifungals (19.7%) and minerals (17.4%), suggesting limited supply or lower demand for these specialized treatments. These disparities highlight potential gaps in VP access, particularly for less common but critical therapies.

### 3.3. Storage Practices and Cold Chain Maintenance of Veterinary Dugs

In this study, the storage areas of 46 veterinary health facilities (*n* = 34 governmental clinics and *n* = 12 private veterinary retailers) were assessed through direct observation using 15 standard criteria that are necessary for implementing drug storage areas. The study found that the design and layout based on standards were satisfied in the majority 26 (76.5%) and 9 (75%) of the governmental and private veterinary drug stores, respectively. About 28 (82.4%) governmental clinics and 8 (66.7%) private drug retailers of stores have separate storage and dispensing areas. From the surveyed 12 private retailers in 11 (91.37%) stores, practices like separating damaged and expired products from usable products and making identification labels such as manufacturing dates and expiry dates visible were implemented. Conversely, cold chain maintenance equipment like refrigerators and ice boxes is available only in 12 (35.3%) governmental clinics and 3 (25%) private retailers. The performance of the storage conditions of the surveyed facilities was presented in Supporting File 1.

**Figure 2:**
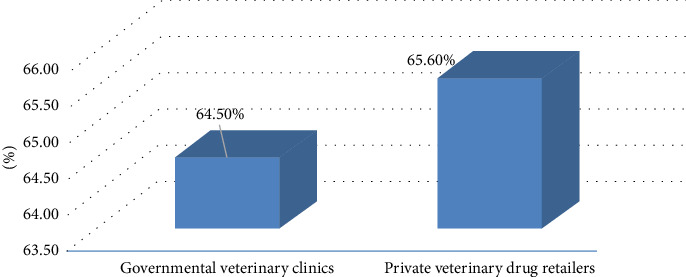
The average compliance to acceptable storage conditions by facility type.

Additionally, a study found that the average performance of surveyed facilities that complied with the criteria for acceptable storage conditions is 64.5% and 65.6% at governmental veterinary clinics and private veterinary drug retailers, respectively (Figure 2).

**Figure 3:**
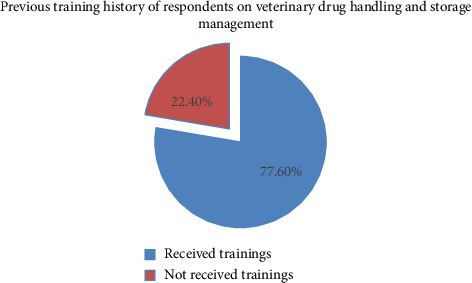
Percentage training history of respondents on drug storage management and handling practices.

Furthermore, the study also found that the majority (77.6%) of respondents had received training on veterinary drug handling and storage conditions (Figure 3).

### 3.4. KAP Assessment on Veterinary Drug Handling and Storage: Insights From Veterinary Professionals

To assess the KAP of veterinary professionals toward the safe handling and storage management of veterinary drugs, by considering the number of veterinary professionals to be employed at the selected facilities, a total of 200 self-administered questionnaires were distributed; from this, a total of 170 veterinary professionals participated in filling out the questionnaires with an 85% response rate.

### 3.5. Knowledge of Veterinary Professionals Toward the Safe Handling of Veterinary Drugs

**Figure 4:**
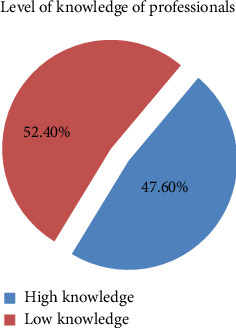
Knowledge of veterinary professional toward the safe handling of veterinary drugs.

The knowledge of veterinary professionals toward the storage management and safe handling of veterinary drugs was assessed using 11 questions. This study found that the majority 116 (68.6%) of the respondents understand that storage conditions can affect the quality of the drug, and 120 (70.6%) of them know that temperature, humidity, and sunlight can affect the drug both during transportation and storage. On the contrary, almost half (48.8%) of the respondents perceived that putting liquid pharmaceutical products on the top shelf is better to handling it safely (Supporting File 2). Furthermore, this study revealed that the majority 89 (52.4%) of the respondents had a low level of knowledge toward the storage management and safe handling of veterinary drugs (Figure 4).

### 3.6. Attitude of Veterinary Professionals Toward the Safe Handling of Veterinary Drugs

**Figure 5:**
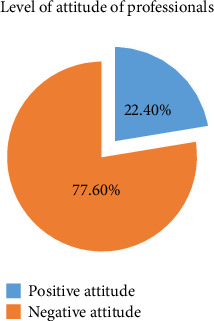
Attitude of veterinary professionals toward the safe handling of drugs.

The attitude of veterinary professionals toward the storage management and safe handling of veterinary drugs was assessed using seven items. This study found that the majority of 90 (52.9%) of the respondents agreed that drug quality has no relationship with drug handling and storage conditions, and about 104 (61.2%) of them disagreed that refrigerators are necessary for veterinary vaccines only. On the contrary, almost more than three/fourths of 107 (62.9%) of the respondents perceived that improper drug storage practice is not the current issue in Ethiopia; hence, there is no need to be wary about it (Supporting File 3). Generally, this study revealed that the majority 132 (77.6%) of the respondents had a negative attitude toward the storage management and safe handling of veterinary drugs (Figure 5).

### 3.7. Practices of Veterinary Professionals Toward the Safe Handling of Veterinary Drugs

The attitude of veterinary professionals toward the storage management and safe handling of veterinary drugs was assessed using seven items. This study found that the majority 75 (44.1%) of the respondents always practiced storing veterinary drugs according to the manufacturer's direction, and a very small 26 (15.3%) of them did not practice this.

**Figure 6:**
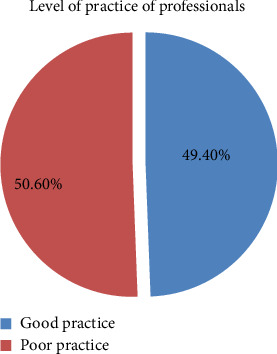
Practice of veterinary professionals toward the safe handling of veterinary drugs.

Additionally, about 19 (11.2%) of respondents never practice referring drug leaflets and handling manuals prior to storing drugs. The study indicated that the majority of 111 (65.3%) always advise their customers and end users on safe drug handling practices (Supporting File 4). Furthermore, a study revealed that almost more than half 86 (50.6%) of the respondents had poor practices toward the storage management and safe handling of veterinary drugs (Figure 6).

## 4. Discussion

The current study focuses on assessing veterinary drug availability, storage conditions, and KAP of veterinary professionals' attitudes toward the safe handling and management of veterinary drugs in and around Nekemte, west Oromia, Ethiopia. According to the principles of the World Health Organization (WHO) and the recommendations of different researchers to provide sustainable healthcare services, drugs should be accessible and available at all times in each service delivery point [36, 37]. However, the current study indicated that the availability of commonly used veterinary drugs at the surveyed facility was not sufficient. This is somewhat similar to the study conducted in SSA, where access to veterinary drugs was far from given and remains an issue for many farmers [15].

This study revealed, among veterinary drugs, that multivitamin is the most widely available drug in 84.8% of facilities, followed by oxytetracycline 20% and oxytetracycline 10%. This finding was lower than the result of the study conducted by Samuel Alebign and Nato Hundesa on the availability and rational use of veterinary drugs in veterinary clinics of Haramaya and Dire Dawa Districts, Eastern Ethiopia, which showed that the availability of oxytetracycline at 20% and oxytetracycline at 10% was 100% [14]. The result of the current study is also lower as compared to the result of the other study conducted by Zeru Hailu, which indicated that oxytetracycline is available in about 90% of the veterinary pharmacy retail outlets [38]. The high availability of multivitamins and oxytetracycline in the current study could be related to their therapeutic use, as oxytetracycline has a broad spectrum and oxytetracycline 20% is particularly used as prophylaxes, and multivitamins are also used as appetizers, and farmers used these drugs for fattening purposes. Anthelmintics are important weapons for the management of helminthic infections in the livestock industry [39]. This study indicated that, in general, the availability of anthelmintic was available in about 72.83% of the facilities; in particular, albendazole 2500 mg was available in the majority 37 (80.4%) of surveyed facilities, followed by Ivermectin injections and albendazole 300 mg, which were present in 36 (78.3%) and 35 (76.1%) of the facilities, respectively. This is different from the study conducted on the assessment of veterinary drug retail outlets in two rural areas of Kwara State, north-central Nigeria, which showed that albendazole was the only type of anthelminthic stocked by the veterinary shops [20]. Conversely, the current study showed that antifungals were available only in 18.3% of facilities at the time of data collection. The low level in the availability of antifungals might be due to the low prevalence of fungal infections in the current study area.

In the current survey, Diminazene aceturate emerged as the most widely available antiprotozoal drug present in approximately 74% of veterinary outlets. In contrast, Isometamidium was available in only 21.7% of facilities, indicating potential understocking that could compromise the effective treatment of protozoal infections in animals. These findings align with the existing literature, which highlights Diminazene as the most commonly distributed trypanocidal drug among local farmers, while Isometamidium is rarely stocked or prescribed [19]. The scarcity of isometamidium, despite its clinical relevance for prophylaxis in Trypanosomiasis, along with the moderate supply of Amprolium and dominance of Diminazene, underlines a critical imbalance in the availability of essential therapies, suggesting gaps in veterinary supply chains, regulatory oversight, and rational distribution of key antiprotozoal drugs across livestock‐producing regions.

Evidence suggests that employing qualified drug storage personnel and providing necessary training are crucial elements to improving the productivity of storage operations [18, 19]. The present study revealed that the three/fourth (77.60%) of study participants had received training on veterinary drug handling and storage management. This is somewhat similar to the result conducted in Adea Berga District, Central Ethiopia, which showed that over 77.6% of the respondents had awareness about the drug store [22]. In contrast, a study conducted on the veterinary drug supply chain in Uganda found that nearly 90% of drug retailers and veterinary drug practitioners lacked specialized training in veterinary medicine handling and storage management [26]. The result was also somewhat different from the result of the study conducted in Ethiopia, which showed that about 57.3% of the study participants had not received on-the-job training in related veterinary drug management, handling, and other related activities. The result is also higher compared to the result of the study conducted in Ethiopia, which indicated that only 22.2% of veterinary drug dispensers have taken training on antimicrobial selection, use, resistance, and resistance containment [38]. This observed difference might be due to the facility's commitment in preparing training programs for their employees.

Veterinary drug storage conditions are regarded as the cornerstone of warehouse management practices, and any defect in the storage area may result in obsolescence, deterioration, spoilage, pilferage, breakage of stock due to excessive overstocking, and even the development of poisonous degradation products that can be hazardous to the patient [16, 40]. The present study revealed that the average adherence to meet criteria for proper storage conditions in veterinary clinics and private drug retailers were somewhat similar (64.5% and 65.6%), respectively, which implies that both the surveyed facilities, governmental veterinary clinics, and private veterinary drug retailers did not meet the criteria for acceptable storage conditions, which is below the acceptable range (80%). Regarding governmental clinics, these results were higher as compared to the study conducted on the VP storage condition in the Amhara region, which found that 48.3% of veterinary clinics met the criteria for acceptable storage conditions [17]. This observed difference may be due to the commitment of governmental bodies and agricultural offices to maintain the storage area of drugs in the current study area. But in relation to private veterinary drug retailers, the storage condition obtained in the current study was lower as compared to the result of a similar study conducted on veterinary drug storage conditions, which showed that meeting the criteria for an acceptable study was about 86.25% (> 80%) [17]. This observed difference in storage performance could be due to the fact that private veterinary drug retailers might be inspected by governmental regulatory bodies and that strict control for the layout and fulfillment of the storage area will be exercised prior to delivering a license. Therefore, in the current study area, the commitment of governmental regulatory bodies might be low to inspecting, controlling, and providing feedback, and supervising veterinary drug retailers. In contrast, the result was somewhat consistent with the findings of the study in the instance of private medical pharmaceutical wholesalers in Gondar, Ethiopia, which indicated that the facilities' storage performance was only about 68.75% [30].

Veterinarian specialists must appropriately oversee and monitor the sensible use of veterinary medications, like the supply, transportation, storage and handling of prescriptions, dispensing, and practices. When using antibiotics, one must adhere to the approved product information, which includes the package leaflet, labeling, and summary of product [22]. The awareness and expertise of livestock producers as well as the knowledge of veterinary medicine dispensers are key factors in the responsible use of veterinary medications. When purchasing veterinary medications, the majority of farmers deal with store employees; as a result, farmers have faith in the shop employees' advice and drug administration techniques. This study revealed that professionals have scored low knowledge, negative attitude, and poor practices (52.4%, 77.6%, and 50.6%) of respondents on the safe handling of and management of veterinary drugs, respectively. This is somewhat similar to the result of the study conducted at the veterinary drug supply chain in Uganda, which indicated that the low level of education of supply chain actors, particularly drug retailers, poor handling of drugs at purchase and administration practices, low enforcement of policies and regulations, and the lack of awareness of stakeholders about policies that regulate drug use [26]. The result of the current study is somewhat consistent with the result of the study conducted in Ethiopia by different researchers in different parts of the country, which revealed a lack of information and enough knowledge about safe handling of drugs and their management, inadequate training, and education of graduates of veterinary medicine, and with the result of another study conducted in Adea Berga District, Central Ethiopia, which showed the majority of them had no enough knowledge about safe handling and its management [22, 41]. The study conducted on the assessment of veterinary drug handling, management, and supply chain in Ethiopia's Afar Pastoral Region found that approximately 63.9% of respondents lacked sufficient knowledge on safe handling and management of veterinary drugs [16].

## 5. Conclusion and Future Needs

This study highlights critical gaps in the availability, storage, and handling of veterinary drugs in surveyed health facilities across Oromia, Ethiopia. Limited access to essential veterinary medications—most notably multivitamins, which were the most commonly available—reflects a significant shortfall in meeting the region's animal healthcare needs. Moreover, substandard storage conditions in both public and private sectors, combined with inadequate KAP among veterinary professionals, raise concerns about the safety and effectiveness of veterinary drug use in clinical practice. To address these challenges, coordinated efforts among the Oromia Regional Livestock and Agricultural Bureau, health facilities, and the Veterinary Drug and Animal Feed Administration and Control Authority (VDFACA) are essential. Improving drug storage infrastructure, strengthening regulatory oversight, ensuring a consistent drug supply, and investing in continuous professional development play a critical role. Additionally, nationwide research is suggested to fully assess the scope of these issues and inform strategic policy interventions for sustainable veterinary healthcare delivery.

## Figures and Tables

**Table 1:** Sociodemographic characteristics of the study participants.

Study participants		*n* (%)
Types of the facility	Government vet clinic	34 (73.9)
	Private veterinary pharmacy retailers	12 (26.1)

Gender	Male	106 (62.4)
	Female	12 (26.1)

Age	18–30 years	49 (28.8)
	31–50 yrs	97 (57.1)
	> 50 years	24 (14.1)

Educational back ground	Diploma	49 (28.8)
	Degree	98 (57.6)
	MSc and above	23 (13.5)

Educational qualification	DVM	26 (15.3)
	Vet. pharmacy	6 (3.5)
	BVS	88 (51.8)

Current position	Advanced animal health	50 (29.4)
	Veterinary clinician	100 (58.8)
	VHI supply and distribution officer	11 (6.5)
	Drug store personnel	9 (5.3)
	Drug dispenser	40 (23.5)
	VLT	10 (5.9)

Work experience	Less than 1 year	20 (17.8)
	1–4 years	72 (42.4)
	5–9 years	60 (35.3)
	Above 10 years	18 (10.6)

*Note: n* (%): Frequency and percentage, Vet: veterinary.

Abbreviations: BVs, Bachelor of Veterinary Sciences; DVM, Doctor of Veterinary Medicine; VHIs, veterinary health inputs; VLT, Veterinary Laboratory Technologies.

**Table 2:** Percentage availability of veterinary pharmaceutical across surveyed health facilities (*n* = 46).

Class of drugs		Availability *N* (%)	Not available *N* (%)
Antibacterials	Oxytetracycline 10%	36 (78.3)	10 (21.7)
	Oxytetracycline 20%	39 (84.8)	7 (15.2)
	Penstrep	32 (69.6)	14 (30.4)
	Sulfonamides	29 (63.0)	17(37)
	Gentamycin	12 (26.1)	34 (73.9)

Anthelminthic	Albendazole 2500 mg	37 (80.4)	9 (19.6)
	Albendazole 300 mg	35 (76.1)	11 (23.9)
	Ivermectin injection	36 (78.3)	10 (21.7)
	Fenbendazole sachet	33 (71.7)	13 (28.3)
	Tetramisole 600 mg	28 (60.9)	18 (39.1)
	Triclabendazole 900 mg	32 (69.6)	14 (30.4)

Antiprotozoal	Amprolium hydrochloride	18 (39.1)	28 (60.9)
	Diminazine aceturate	34 (73.9)	12 (26.1)
	Isometamidium	10 (21.7)	36 (78.3)

Antiseptics	Alcohol 70%–96%	38 (82.6)	8 (17.4)
	Iodine solutions	36 (78.3)	10 (21.7)
	Potassium permanganate	21 (45.7)	25 (54.3)

Antifungal	Ketoconazole	9 (19.7)	37 (80.4)

Mineral	Copper sulfate	8 (17.4)	38 (82.6)

Vitamin	Multivitamin	39 (84.8)	7 (15.2)

Indigestion powder		16 (34.8)	30 (65.25)

## Data Availability

The data that support the findings of this study are available from the corresponding author upon reasonable request.

## Nomenclature

AMR: Antimicrobial resistance
AMU: Antimicrobial use
EAA: Ethiopian Agricultural Authority
KAP: Knowledge attitude and practices
LIAT: Logistic indicator assessment tool
VDFACA: Veterinary drug and feed administration control authority
VPs: Veterinary pharmaceuticals
WHO: World Health Organizations

## Supporting Information

Supporting Information 1 Supporting File 1: The performance of meeting acceptable storage conditions of the surveyed facilities.

Supporting Information 2 Supporting File 2: Knowledge of veterinary professional toward the safe handling of veterinary drugs.

Supporting Information 2 Supporting File 3: Attitude of veterinary professional toward the safe handling of veterinary drugs.

Supporting Information 4 Supporting File 4: Practices of veterinary professionals toward the safe handling of veterinary drugs.
