# Supplementary material for: Assessment of Veterinary Drug Availability, Storage Conditions, and Handling Practices in and Around Nekemte Town, Southwestern Oromia, Ethiopia
Source: Vet Med Int. 2025 Sep 3;2025:7813053. doi: 10.1155/vmi/7813053 (PMC12422859; doi:10.1155/vmi/7813053)
Supplement: Supporting Information 1 — Supporting File 1: The performance of meeting acceptable storage conditions of the surveyed facilities. [file 7813053.f1.docx]

**Supplementary File 1:** The performance of meeting acceptable storage conditions of the surveyed facilities (n=46)

| Item descriptions | Response category N (%) | | | | | |
| --- | --- | --- | --- | --- | --- | --- |
|  | Governmental clinic (n=34) | | | Private pharmacy (n=12) | | |
|  | Yes N (%) | | No N (%) | Yes  N (%) | | No  N (%) |
| Availability of separate storage and dispensing area | 28 (82.4%) | 6 (17.6%) | | | 8 (66.7%) | 4(33.3%) |
| Availability of shelf and pallets in storage area | 25(73.7%) | 9(26.5%) | | | 10 (83.3%) | 2(16.7%) |
| Storage area is designed and layout based on standards (shelf with 20 cm away from the walls , palate with at least 10 cm off the floor and length of the rack with at least 2. 7 m) | 26 (76.5%) | 8 (23.5%) | | | 9 (75%) | 3(25%) |
| Identification labels, manufacturing dates and expiry dates are visible | 25 (73.5%) | 9(26.5) | | | 11 (91.7%) | 1(8.3%) |
| Cartons and products are in good condition, not crushed due to mishandling | 23(67.6 %.) | 11(32.4) | | | 9 (75%) | 3(25%) |
| Cartons and products are protected from water and humidity | 26 (76.5%) | 8 (23.5%) | | | 8 (67.7%) | 4(33.3%) |
| Products are protected from direct sunlight | 25(73.5) | 9(26.5%) | | | 7(58.3%) | 5(41.7%) |
| The storage area is visually free from harmful insects and rodents | 22(64.7%) | 12(35.3%) | | | 9(75%) | 3(25%) |
| The facility makes it a | 24(70.6%) | 10(29.4%) | | | 11(91.7%) | 1(8.3%) |
| The current space and organization are sufficient for existing products | 21(61.8%) | 13(38.2%) | | | 7(58.3%) | 5(41.7%) |
| The roof is maintained in good condition to avoid sunlight and water Penetration | 23(67.6%) | 10(29.4%) | | | 9(75%) | 3(25%) |
| The storeroom is maintained in good condition (clean, all trash removed, sturdy shelves, organized boxes). | 14(41.2%) | 20(58.8%) | | | 4(33.3%) | 8 (66.7%) |
| Fire safety equipment and wall thermometer are available | 23(67.6%) | 11(35.3%) | | | 8 (66.7%) | 4(33.3%) |
| Cold chain maintenance equipment like refrigerator and ice box are available | 12(35.3%) | 22(64.7%) | | | 3(25%) | 9(75%) |
| Availability of alternative power for the outages of electricity | 12(35.3%) | 22(64.7%) | | | 5(41.7%) | 7(58.3%) |
| Average | 64.5 | 36.5 | | | 65.6 | 34.4 |
