# Supplementary material for: Assessment of Veterinary Drug Availability, Storage Conditions, and Handling Practices in and Around Nekemte Town, Southwestern Oromia, Ethiopia
Source: Vet Med Int. 2025 Sep 3;2025:7813053. doi: 10.1155/vmi/7813053 (PMC12422859; doi:10.1155/vmi/7813053)
Supplement: Supporting Information 2 — Supporting File 2: Knowledge of veterinary professional toward the safe handling of veterinary drugs. [file 7813053.f2.docx]

**Supplementary File 2.** Knowledge of veterinary professional towards the safe handling of veterinary drugs (n= 170)

| Specific items for Knowledge to drug handling and storage management | Response category yes n (%) | |
| --- | --- | --- |
|  | Yes n (%) | No n (%) |
| Storage conditions can affect the quality of the drug | 116 (68.6%) | 53 (31.4%) |
| Drugs quality is not reduced before expired date | 118 (69.4%) | 52 (30.6%) |
| drug shelf life will reached if drugs are not handle properly | 104 (61.2%) | 66 (38.8%) |
| Vaccine needs special storage condition than other veterinary drugs | 117 (68.8%) | 53 (31.2%) |
| Ventilation is necessary everywhere for all types of veterinary drugs | 139 (81.8%) | 31 (18.2%) |
| Temperature, humidity and sunlight can affect the drug both  during in transportation and storage area | 120 (70.6%) | 50 (29.4%) |
| Store at refrigerator is storing drug products at the range 2°C and 8°C | 119 (70.0%) | 51 (30.0%) |
| Store at cool place is storing drugs at a temperature not exceeding 8°C | 98 (57.6%) | 72 (42.4%) |
| Depending on climatic conditions of the area Store drugs at room  temperature is storing it at the range of 15°C and 30°C | 118 (69.4%) | 52 (30.6%) |
| Putting liquid pharmaceutical products at the top  shelf is better to handling it safely | 87 (51.2%) | 83 (48.8%) |
| Putting Heavy pharmaceutical products at the bottom shelf is  better to handling it safely | 112 (65.9% | 58 (34.1%) |
