# Supplementary material for: Assessment of Veterinary Drug Availability, Storage Conditions, and Handling Practices in and Around Nekemte Town, Southwestern Oromia, Ethiopia
Source: Vet Med Int. 2025 Sep 3;2025:7813053. doi: 10.1155/vmi/7813053 (PMC12422859; doi:10.1155/vmi/7813053)
Supplement: Supporting Information 2 — Supporting File 3: Attitude of veterinary professional toward the safe handling of veterinary drugs. [file 7813053.f3.docx]

**Supplementary File 3:** Attitude of veterinary professional towards the safe handling of veterinary drugs

| **Specific items for Attitude to drug handling and storage management** | **Response category** | |
| --- | --- | --- |
|  | **Disagree** | **Agree** |
| Drug quality has no relationship with drug handling and  storage conditions | 80 (47.1%) | 90 (52.9%) |
| Proper storage condition can extend the expire date of drugs | 78 (45.9%) | 92 (54.1%) |
| Refrigerator is necessary to veterinary vaccines only | 104 (61.2%) | 66(38.8%) |
| It is okay storing expired drugs with together usable  products as a far as the storage area is sufficient enough | 98 (57.6%) | 72 (42.4%) |
| Maintaining the good storage conditions of drugs  is the responsibility of store personnel/keepers only | 92 (54.1%) | 78 (45.9%) |
| Currently improper drug storage practice is not the  issue of Ethiopia hence no need worry about it | 63 (37.1%) | 107 (62.9%) |
| Do you have enough knowledge on safe handling, storage  and management of drugs starting from acquisition to end user | 79 (46.5%) | 91(53.5%) |
