# Supplementary material for: Assessment of Veterinary Drug Availability, Storage Conditions, and Handling Practices in and Around Nekemte Town, Southwestern Oromia, Ethiopia
Source: Vet Med Int. 2025 Sep 3;2025:7813053. doi: 10.1155/vmi/7813053 (PMC12422859; doi:10.1155/vmi/7813053)
Supplement: Supporting Information 4 — Supporting File 4: Practices of veterinary professionals toward the safe handling of veterinary drugs. [file 7813053.f4.docx]

**Supplementary File 4:** Practices of veterinary professionals towards the safe handling of veterinary drugs (n= 170)

| **Specific items for practices to drug handling and storage management** | **Response category** | | |
| --- | --- | --- | --- |
|  | **Always** | **Sometimes** | **Never** |
| In our clinic storage area; drugs are stored according to manufacturer’s direction | 75 (44.1%) | 69 (40.6%) | 26 (15.3%) |
| I use Special vehicle than Public transport during drug transportation | 89 (52.4%) | 64(37.6%) | 17 (10.0%) |
| I refer drug leaflets and handling manuals prior to storing drugs | 73 (42.9%) | 78 (45.9%) | 19 (11.2%) |
| In our store drugs are arranged in First Expire First out (FEFO) (The early expiry retrieve first) principle | 61(35.9%) | 83(48.8%) | 26 (15.3%) |
| In practice Flammable chemicals like alcohol are stored separately and in specialized area | 74(43.5%) | 83(48.8%) | 13 (7.6%) |
| I practice packaging of drugs while selling to clients even I sell a single bolus or vial | 80(47.1%) | 66(38.8%) | 24 (14.1%) |
| I advise my customers and end users on safe drug handling | 111 (65.3 %) | 59 (34.7%) | 0.00 |
